# Emerging Technology in Promoting Physical Activity and Health: Challenges and Opportunities

**DOI:** 10.3390/jcm8111830

**Published:** 2019-11-01

**Authors:** Zan Gao, Jung Eun Lee

**Affiliations:** 1 School of Kinesiology, University of Minnesota, 208 Cooke Hall, 1900 University Ave. SE, Minneapolis, MN 55455, USA; 2 Department of Applied Human Sciences, University of Minnesota, Duluth, MN 55812, USA; junelee@d.umn.edu

Sedentary behavior has been identified as one of the major causes of many chronic diseases such as cardiovascular disease, stroke, cancer, type 2 diabetes, and obesity [1]. Emerging technology plays a complex role in sedentary behavior—very much like a double-edged sword. On one side, some emerging technologies (e.g., sedentary video games and computer games) have contributed to the epidemic of sedentary behavior and physical inactivity. On the other side, other innovative technologies have been increasingly utilized to promote physical activity (PA) and health [2,3]. For example, newly emerging technologies such as mobile device applications, health wearable devices, and active video games have been adopted to promote health [4,5,6,7,8]. As technology becomes an ever more prevalent part of everyday life and population-based health programs seek new ways to increase life long engagement with PA, so the two have become increasingly linked.

This special issue titled “*Emerging Technology Applications to Promote Physical Activity and Health*” has been published in *Journal of Clinical Medicine* in 2018. It attempted to offer a thorough, critical examination of emerging technolo gies in PA and health promotion, considering technological interventions in different contexts (communities, clinics, schools, homes, etc.) among various populations, exploring the challenges of integ rating technology into PA promotion and offering solutions for its implementation. This special issue aimed to occupy a broadly positive stance toward interactive technology initiatives and, while discussing some negative implications of an increased use of technology, offered practical recommendations for promoting PA through various emerging technologies, including but not limited to: exergaming (active video games); social media; mobile device apps; health wearables; mobile games, augmented reality games, global positioning and geographic information systems (GPS/GIS); and virtual reality.

In detail, newly databased findings and systematic reviews have been presented from 14 studies, which took place in various countries and regions of the world. These studies attempted to: (1) examine effects of exergaming on children’s PA and health outcomes; (2) explore the benefits of applying mobile apps, wearable devices and social media; (3) test the validity of activity monitors in assessing PA in various settings; (4) investigate the relationships among environmental factors and sedentary behaviors; (5) explore the application of augmented reality and virtual reality games in real-world settings; and (6) provide directions for future research and practice in the promotion of PA and health through emerging technology.

## 1. Exergaming on Children’s PA and Health Outcomes

Exergaming has been praised as an innovative and useful medium by which to promote PA in various populations, especially among children. In this review, Benzing and Schmidt [9] explored the present status and future of exergaming implementation in the health and education field in diverse populations. The researchers reported examples of the strengths of exergaming in providing opportunities for PA and health promotion, such as increased PA enjoyment, the ability to reach specific populations (e.g., children with attention deficit hyperactivity disorder), and individualization. These authors reported, however, that there were some weaknesses, such as technical restrictions and the inability to sustain the programs over the long-term. These weaknesses, unfortunately, will threaten traditional exercise and may translate into increased screen time. Quan et al. [10] investigated patterns of PA behaviors in first and second grade children while engaging in exergaming. The researchers used accelerometry to measure the PA behaviors and examined four sessions at random from the 27 exergaming sessions. The researchers found that the average percentage of time spent in sedentary behavior was 47.2%, followed by light PA (LPA) 32.9% and moderate-to-vigorous PA (MVPA) 19.9% in each 30-min exergaming session. Additionally, it was shown that there were no gender differences in these PA behaviors, which altogether indicates that exergaming may be a good means to promote children’s LPA and MVPA among both sexes. Ye et al. [11] examined the effect of a nine-month combined physical education (PE) and exergaming program on children’s motor skills, namely object manipulation and locomotor skills (adapted from the Test of Gross Motor Development-2) and health-related fitness compared to a traditional PE program. While children in the traditional PE group demonstrated significantly higher cardiorespiratory fitness than the intervention group, the decrease in body mass index (BMI) of children in the intervention group was significantly different from the increased BMI of children in the traditional group. In terms of fitness, musculoskeletal fitness scores for the intervention group were significantly higher compared to that of the traditional group. The findings suggest that incorporating exergaming as part of the PE curriculum over an academic year may improve children’s muscular strength and BMI.

## 2. Applications of Mobile Apps, Wearable Devices, and Social Media

Lee and colleagues [12] investigated the effect of a tailored Mammogram mobile app (mMammogram) on breast cancer screening behavior in Korean American immigrant women. In this qualitative study, the authors found three themes: (1) the women had increased knowledge on the necessity for the breast cancer screening and screening procedures; (2) health navigators are needed to support these women to receive benefits from the healthcare system; and (3) mMammogram needs to be further developed so it could be downloaded onto various models of smartphones and tablets. The researchers concluded that a culturally-tailored health navigation service is important in overcoming the barriers to healthcare accessibility and supporting the immigrant population in the adoption of appropriate health screening behaviors. Pope et al. [13] examined the effectiveness of a 10-week combined smartwatch and social media intervention on health outcomes among breast cancer survivors. The intervention group wore Polar smartwatches to track PA and joined a Facebook social group to receive twice-weekly PA-related tips. The comparison group only joined a separate Facebook group with the same PA tips. The researchers reported that both groups similarly increased LPA, MVPA, energy expenditure, and steps over time. Interestingly, the comparison group showed improved psychological variables (e.g., PA-related social support and decreased barriers) while the intervention group demonstrated decreased social support and no change in barriers. Harris and Chen [14] examined the impact of four-week PA breaks using Fitbit on fifth graders’ real-time PA and cardiovascular fitness. Students were assigned to one of the following three groups: (1) Fitbit-O: wore Fitbit as a self-monitoring tool; (2) Physical Activities Engaging the Brain plus Fitbit Challenge (PAEB-C): engaged in daily six-min guided PA breaks during class in addition to wearing the Fitbit; and (3) control group. The results showed that the PAEB-C group reported a significantly higher number of daily real-time steps and more time spent in PA compared to the Fitbit-O group. Furthermore, there were significant differences in the fitness score between the Fitbit-O and the control and also between the PAEB-C and the control group, but no significant differences were found between the two Fitbit groups.

## 3. Assessing Validity of Wearable Devices 

The preschool years are a critical period to develop cognitive function and engage in various PAs. Quan et al. [15] explored the relationship between Chinese preschoolers’ seven-day PA measured via accelerometers and their cognitive functions. The correlation between PA and cognitive functions were not statistically significant in girls. However, the researchers found that boys’ LPA was significantly related to their cognitive functions. Furthermore, when total PA (i.e., sum of LPA and MVPA) was entered in the model, replacing LPA and MVPA, it was associated with cognitive function, solely in boys. The authors suggested these gender-specific results may have been due to a mediating effect of cardiorespiratory fitness.

Exergaming has been a popular means to engage children in PA in an enjoyable way. However, accurate assessment of PA in an exergaming setting is limited. Zeng et al. [16] examined the reliability of ML-1000 pedometers and GT3X+ accelerometers in assessing children’s PA during a 30-min exergaming session. The assessment was taken during 21 exergaming sessions, and the intraclass correlation coefficient results indicated low reliability in accelerometers and pedometers in the assessment of PA during the sessions. Further, the results of hierarchical linear modeling, which controlled the learning effect of the pedometer, also pointed to the intraclass correlation coefficient results. Additionally, no positive relationship was found between steps per minute and time spent in MVPA. In another study, Hwang and colleagues [17] investigated the placement of accelerometers and appropriate epoch length for adults’ PA assessment during exergaming. Forty-seven young adults wore both wGT3X+ and GT9X accelerometers on both the wrist and the hip. The findings from intraclass correlation coefficients and Bland-Altman plots indicated a good inter-monitor agreement in steps and activity counts when worn on both the hip and the wrist. In terms of epoch length, a one-second epoch while worn on the hip showed the most accurate match with exercise intensity measured by a heart rate monitor. The researchers also found that that there was better accuracy of step counts and PA estimation for both models when the accelerometers were worn at the hip.

## 4. Relationships among Environmental Factors and Sedentary Behaviors 

Past literature has reported the association between air pollution and physical inactivity. In this study, Ma and colleagues [18] examined the relationship between air quality and sedentary behaviors in Chinese adults. Time spent in sedentary behavior was measured by wrist-worn accelerometers, and the association between the air quality and sedentary behavior was adjusted for demographic variables, BMI, wake time, and weather-related variables. It was found that Chinese adults spent approximately 573 min per day sedentary. The results also revealed that on days with good air quality, adults spent approximately 20 min less in sedentary behavior compared to days with more polluted air. Additionally, higher concentrations of fine particulate matter were also related to longer sedentary time than other days with lower concentrations. In another study, Hsueh and colleagues [19] investigated associations of older adults’ perceived environmental factors with self-reported leisure-time PA and screen time. Perceived environmental factors consisted of 11 attributes, such as density, accessibility, safety, presence of sidewalks, and aesthetics. The results showed that good accessibility to shops and public transportation and good connectivity of streets were negatively associated with screen time of more than two hours. The researchers also revealed that six of the 11 environmental attributes were associated with achieving 150 min per week of leisure-time PA. The six attributes were good access to shops, public transportation, recreational facilities, seeing people being active, good aesthetics, and the presence of a destination. 

## 5. Applications of Augmented Reality and Virtual Reality Games

Virtual reality therapy has been increasingly used in treating people with mood disorders. However, the effect of virtual reality-based exercise on anxiety and depressive symptoms are not well documented. Zeng et al. [20] conducted a preliminary systematic review on the association between virtual reality exercise and anxiety and depression. The findings indicated that virtual reality-based exercise was effective for improving moods related to anxiety and depression. Specifically, following virtual reality exercise, positive effects, such as energy and enjoyment, increased while the negative effect (i.e., tiredness and tension) was reduced. However, more evidence with stronger research designs is needed to fully support the effectiveness of virtual reality exercise on the treatment of anxiety and depression.

In a randomized trial, Ryu and colleagues [21] examined whether an immersive virtual reality game that allows pediatric patients to experience a preoperative procedure would alleviate anxiety in children going in for surgery. Children in the virtual reality gamification group played one 5 min game in which children experienced the preoperative process and anesthesia induction through a virtual world one hour before going into the operating room. The findings indicated that the gamification group showed significantly lower preoperative anxiety compared to the children in the control group. Additionally, children in the gamification group demonstrated better compliance during the induction of anesthesia. It was concluded that the combined effect of gamification and virtual reality exposure might effectively reduce anxiety in pediatric patients. In another study, Sahin and colleagues [22] assessed the safety and potential negative effects of novel augmented reality smart glasses that are used as a communication aid for people with autism. Each participant was accompanied by their caregivers, and participants and caregivers interacted with each other through a series of gamified experiences through the glasses. Following the interaction, structured interviews were conducted, and it was reported that 88% of the users and 100% of caregivers reported no minor negative effects. Concerns regarding dizziness, eye strain, and nasal bridge discomfort were reported, but these were mild. Additionally, most users and their caregivers did not have any design issues regarding the system. The only design concern raised was the smart glasses becoming warm to the touch during use.

## 6. Emerging Technology for PA Assessment and Promotion 

Technology is embedded in our society and is changing our lives in positive and negative ways. Indeed, emerging technologies have left no scientific field untouched, including the field of PA and health. As stated a number of times within this editorial, however, technology is like a double-edged sword when viewed through the lens of PA and health promotion. In fact, these emerging technologies have entered into homes, schools, communities, and other avenues, and have been very popular among various populations [23,24,25,26,27,28,29,30,31]. For example, nowadays, smartphones not only allow us to regularly communicate with others through phone calls and text messages but also let us monitor our health status and monitor PA behaviors via multiple health-oriented apps [32]. Healthcare professionals can play a valuable role in the selection and integration of emerging technology. This section will equip the readers with the requisite knowledge base regarding the use of emerging technology for PA assessment and promotion. We hope that such knowledge will promote further integration of emerging technology into PA and health by scholars and healthcare professionals alike. 

### 6.1. Emerging Technology for PA Assessment 

Technology is constantly changing our living environment and lifestyles. Approximately two decades ago, PA questionnaires were replaced with health wearables (e.g., pedometers) that measured PA and tracked and calculated human movement patterns for analyses. Particularly, the most popular electronic devices, such as pedometers, accelerometers, and heart rate monitors, provide details of PA measures, including but not limited to steps, estimated distance, energy expenditure, time in different intensity levels of PA, and heart rate [33]. Beyond their measurement attributes, these devices also play an important role in providing feedback to clients, which can act to motivate these individuals to participate in more PA. 

With the rapid development of technology, accelerometers capable of assessing PA and sedentary behavior are now appearing in smartphones and GPS devices. While technology advancement offers opportunities for the public to engage in sedentary behavior, the integration of accelerometers into a greater number of electronic devices is providing opportunities for professionals to objectively assess PA and sedentary behavior to address various health issues. In fact, the extant electronic devices are attractive for healthcare professionals in that they have achieved greater ease of use, greater precision, and greater scope (i.e., data of various sources from one device) by integrating innovative emerging technologies, such as mobile device apps, GPS units, and health wearables. With this information, knowing where, when, and how PA occurs allows for greater understanding of PA patterns and, ultimately, allowing for more effective PA behavioral changes [34]. 

Recently, many mobile devices (smartphones and tablets) and fashion accessories (belt, necklace, bracelet, and rings) have embedded GPS and accelerometer technologies, which are being used to identify and promote PA participation as well as understand naturalistically-occurring activities [35]. Some smart devices are even taking advantage of the camera function of mobile devices. For example, SenseCam, a wearable digital camera that takes photos automatically without user intervention, along with mobile apps and other visual devices, can provide details of PA contexts and an individuals’ lifestyle.

Furthermore, technology advancement has included the development of multiple sensor systems, enabling better identification of previously non-identifiable PA (e.g., bench press, stair climbing) through pedometers or accelerometers alone. Presently, the vast majority of monitors (i.e., Actigraph), devices (i.e., Fitbit Versa Smart Watch), or apps (i.e., MapMyFitness, Keep) are able to send data to an individual’s account and relevant communities via remote servers. This invites the further embrace of online technology and PA promotion websites such as Stickk.com that let individuals make public contracts visible to other users and also utilize monetary incentives to promote PA behavioral changes. Additionally, technological advancements like crowdsourcing (i.e., applies the masses, or crowds, of individuals using the Internet, social media, and smartphone apps) allows for input from a large base of diverse users that can help to identify and improve infrastructure for PA.

Overall, although validity testing is warranted when applying emerging technologies in assessing and evaluating PA behavior [36,37], it is imperative and noteworthy for scholars and healthcare professionals to embrace new technologies given the fact that: (1) emerging technologies may seamlessly elevate our abilities to analyze PA patterns; (2) the ease of use and transferability can significantly impact large populations from a longitudinal perspective; (3) emerging technologies can improve the ongoing, systematic process evaluation of any intervention program given their real-time data sharing capabilities; (4) big data can be data- or text-mined (e.g., PA data, online posts). PA data will be better accessed with improved algorithms and mathematical models; and (5) the increasingly blurred boundaries between PA assessment and intervention (e.g., self-tracking PA) might require a reevaluation of the traditional scientific model to design and evaluate these types of studies [35].

### 6.2. Emerging Technology in PA Promotion

As previously stated, researchers and healthcare professionals have taken advantage of emerging technology available in PA promotion. However, it should be noted that some emerging technologies such as GPS/GIS have only been used to measure and track PA behavior, with few interventions of this type having the objective of promoting an individual’s PA behaviors. Fortunately, the introduction of persuasive technology shows great potential for emerging technology applications in PA promotion. More specifically, persuasive technology refers to a technology that is designed to change individuals’ attitudes or behaviors through persuasion and social influence, but not through coercion [38]. Research involving persuasive technology focuses primarily on interactive and computational technologies such as video games, desktop computers, the Internet, and mobile devices, yet it also incorporates behavioral theories and human-computer interactions [39]. Indeed, persuasive technology is designed with the goal of changing a particular aspect of human behavior, including PA behavior, in a predefined way in non-commercial domains. 

Some emerging technologies play key roles in persuading individuals to engage in greater PA participation in that: (1) they serve as the tool (e.g., recording heart rates and calories); (2) they are linked to social media (e.g., synchronize exercise app data to Facebook); and (3) they offer social interaction (e.g., Kinect Just Dance 2019 allow for dancing with another person from the online community). The applications of such technology have been increasingly seen in our daily lives. For example, Just Dance, a type of active video game with dance movement, provides scores and instant feedback to users for each of their movements while allowing users to compete with others simultaneously on-site or online. Most importantly, this technology’s interactive feature captures users’ attention so that they play the dance game without knowing that they are actually exercising [34].

Per Dominic et al. [39], emerging technology is generally considered a tool so far, and the technology use alone may not lead to ideal persuasion and expected intervention outcomes. Changing behavior is never an easy task. Therefore, it is critical to integrate emerging technology and behavioral theories to promote PA among various populations. In fact, a number of behavioral theories, including the Social Cognitive Theory and Theory of Planned Behavior, have been widely utilized in some PA interventions involving emerging technology. 

In addition, it is important to recall that multiple technologies have now been integrated into singular apps. Utilizing the power of each technology, combining the technological units provides incredible power for users. Currently, PA assessments and delivery of interventions are available on singular apps. The aforementioned MapMyFitness, Xbox Kinect, and Apple watch are such applications. More research is warranted using these multi-technologies devices.

## 7. Opportunities and Challenges

Emerging technologies bring exciting or even stunning opportunities for the promotion of PA and health. Yet, not all contributions from emerging technologies have been positive. The emerging field of PA and health, coupled with the rapid development of technology, has presented both challenges and opportunities that deserve further considerations for researchers and healthcare professionals. According to King et al. [40] and Gao [34], challenges and opportunities can be classified into five categories: (1) data collection and data expansion; (2) technical considerations; (3) areas for “bridging the gap”; (4) privacy protection, and (5) Internet of things. 

### 7.1. Data Collection and Data Expansion

While processing data gathered from emerging technologies, a number of challenges have emerged. First, there has been a lack of big data analysis despite the potentially large amount of PA data that can be retrieved from multiple emerging technologies (e.g., PA data, posts on social media, GPS/GIS data) to improve population health. Thus, great opportunities exist to deal with big data via improved mathematical models and computer algorithms. Another potential challenge to the integration of emerging technology is incorporating big data analysis. Simply put, big data analysis requires the use of innovative methods to obtain, accumulate, and study the rapidly accruing data existing in different formats (e.g., audio, video text) within our high-tech world [41]. Second, there has been little use of crowdsourcing data in the PA field. Further, many emerging technologies are not yet capable of capitalizing on the ubiquity and heterogeneity of potential environmental data sources or at employing crowdsourcing to evaluate and manage large datasets in an effort to improve public health. As such, crowdsourcing data in PA and health is an emerging field. For example, researchers started to use crowdsourcing to complete PA evaluation and surveillance (Amazon Mechanic Turk; www.mturk.com). It appears promising to take advantage of crowdsourcing data in promoting PA in the future. Third, there is still a lack of sufficient PA data. Training and recruiting more researchers to study PA and emerging technologies is strongly encouraged. Finally, little understanding of person–environment interactions in studies utilizing emerging technologies has been seen. It is, therefore, warranted to increase the number of studies that investigate different dimensions of an individual’s personal attributes (e.g., self-esteem, attitudes, cognition, weight status) and environments (e.g., social environment, physical environment).

### 7.2. Technical Considerations

Technology is rapidly developing at an amazing speed, thus offering numerous challenges and opportunities is ahead of us. First, a challenge exists in an up-to-date manner with the latest technological advancements. Nowadays, it is not uncommon for researchers to find their recently purchased products become outdated after a short period of time. King et al. [40] suggested the centralization of technological resources for researchers with links, critiques, etc. It is also recommended to partner with companies in the public sector to begin developing and testing emerging technologies. Second, it is noteworthy that a virtual exercise advisor has now been applied in PA promotion. Given the major potential of this type of interactive technology, it is advisable for healthcare professionals and researchers to solicit small business research grants to develop cutting-edge technologies for PA promotion. Third, augmented reality games (e.g., Pokémon Go) have successfully gained attention from users in recent years. These reality games are attractive as they integrate the physical and virtual worlds into one interface on mobile devices, particularly the apps of smartphone devices. Of note for healthcare professionals, augmented reality games require users to walk around and explore their local surroundings; hence, increasing PA participation. However, physical harm may occur, such as playing these games while walking or driving. Playing the games also increases economic burden as a result of in-app purchasing and heavy data usage, and potentially leads the users to inappropriate or dangerous areas. In addition, the geo-locating feature embedded within some games can result in crime (e.g., criminals using the “lure” function of Pokémon Go). It is, therefore, imperative to offer the safety guidelines for users of such games. Fourth, the cost of recent technology and relevant equipment and supplies can present an obstacle. In many cases, researchers provide subjects with required equipment and supplies, assuming the investment will increase consistency in intervention across participants and decrease potential barriers to participation and adherence. Yet, it can be quite expensive to offer lots of devices to a large population at one time. To deal with this issue, researchers can solicit research funds and industry donations for technology-based PA promotion programs. Researchers can also enroll participants in cohorts to decrease the number of devices needed. For some programs, it is possible to request that participants cover the costs (e.g., when using a smartphone and its apps as intervention strategies)—potentially providing a more contextually relevant evaluation of the intervention program. Additionally, it is challenging to secure funds for longitudinal data collection. Also, challenges exist due to the discrepancies in access to emerging technology among individuals from different socioeconomic status. It is suggested to locate and use publicly available technological resources or to employ widely used low-fee mobile devices, such as smartphones, for PA promotion [34]. 

Advances in emerging technology also raise cross-technology issues. In some cases, health wearable or mobile devices incorporate several technologies, such as GPS, accelerometers, cameras, gyroscopes, light and sound sensors, and even physiological sensors for assessing electrocardiography and heart rate, to better understand correlates and determinants of PA behavior. Recently, platforms integrating multiple sensors have been developed to explain complex phenomena that fuse data simultaneously from one or more sensors and one or more behaviors (e.g., tracking both PA and diet data like MapMyFitness and Keep). In fact, this type of app may provide detailed contextual information on PA environments on a sizable geographic scale [42]. Indeed, the Personal Activity Location Measurement System, another fully integrated instrument, can provide estimated PA energy expenditure based upon data collected with accelerometers and heart rate monitors along with location data from GPS data loggers [43]. Therefore, it seems the next step in this field is to more fully integrate data from motion sensors (e.g., accelerometer), contextual sensors (e.g., GPS), and physiological sensors, while also including self-reported indicators of health as well (e.g., perceived PA environment, self-efficacy to adhere to an exercise program). As a result of this integration, researchers will be capable of providing a deeper understanding of the interactive effects of contextual- and individual-level factors on PA behavior. That is, the following two issues need to be kept in mind when considering cross-technology integration. First, smartphones typically run out of battery quickly when GPS is utilized. The alternative options would be to use power banks, if possible, or use smartwatches or sports bands with associated app functionality. Second, few studies have employed crowdsourcing to evaluate and manage large datasets in an effort to promote PA and health. Yet, crowdsourcing data in PA and health is the newest and among the most powerful tools presently at researchers’ disposal. It is warranted to take advantage of crowdsourcing data in promoting PA and health in the future. 

### 7.3. Areas for “Bridging the Gap”

To bridge the gap between research on the use of emerging technologies to promote PA in the real world, researchers face a number of challenges as below. First, experts from various disciplines need to develop and implement technology applications to improve health in field-/clinic-based settings. Many times, some well-functioning applications are designed by computer scientists while neglecting the needs of clients, which leads to little or no intervention impact. Hence, to design and implement an effective technology application for PA promotion, it is crucial to recruit researchers and practitioners from different disciplines, including PA specialists, computer scientists, and health practitioners, as well as consulting the end-users. Once more, it is also encouraged to form institutional collaborations with initiatives and monetary incentives. Notably, with the increasing application of the Social Ecological Model, the built environment inevitably becomes an indispensable component of PA promotion. As such, partnering with experts across disciplines (e.g., PA, planning, transportation, technology) is also encouraged. Second, with the integration of electronic devices, mobile devices, and apps, bridging the gap between PA assessment and intervention becomes a challenge for researchers. Thus, working with all stakeholders, including clients, to identify leverage points for behavior change and integrate everyday PA data seamlessly into the lives of participants to promote behavior change strategies will be important in the years to come. Additionally, to effectively implement technology-based PA promotion, it is imperative to understand the community and organizational systems, as well as all other stakeholders’ needs and interests. For example, in school-based PA programs, technology can offer more choices to students. Children may be able to choose from various exergames, such as Wii Switch Sports, Kinect Just Dance, iDance, and Dance Dance Revolution, and other games. This increased autonomy acts as a motivational tool to get players active and improve class participation. However, it is quite challenging to implement such programs at schools due to a lack of support from school administration, space limitations, curricular conflicts, and budget shortages [44]. 

### 7.4. Promote and Model Digital Citizenship

Emerging technologies that continuously collect data regarding PA behavior as well as other social and environmental aspects related to PA invite several important ethical considerations for researchers in the areas of anonymity, privacy, and participant informed consent. Therefore, researchers should develop standardized protocols for privacy protection. Many concerns are present when it comes to the digital realm and its history with the undermining of anonymity. Conceivably, the largest undesirable feature for the use in PA promotion of emerging technologies is the confidentiality of health information while using these new technologies [45,46]. It is critical that researchers remain ethically concerned about the issues of privacy, participant consent, and anonymity as it pertains to emerging technologies that are constantly collecting statistics on behavioral as well as environmental and social characteristics.

In addition, PA research using self-tracking mobile devices and other emerging technologies raises new issues regarding the need to get informed consent from participants. More specifically, if researchers collect PA data over a long period of time or re-use the data in successive experiments, questions arise regarding whether they still need to obtain consent from subjects. Traditionally, informed consent has generally been used for time-limited studies where assessments occurred infrequently. As such, protocols may need to be changed if researchers collect daily PA data from participants using emerging technology. Therefore, it is recommended to fully inform participants of the multiple uses of their data and ensure the participants that the data will be destroyed upon completion of a study. It is important to note that ethical issues pertinent to new forms of digital data have been investigated since the inception of the Internet. Currently, however, Institutional Review Boards hold different perspectives concerning whether issues related to the collection of data via emerging technologies are unique, and thus, there is a need to develop formal universal guidelines to address these issues [47].

### 7.5. The Internet of Health Things

A handful of emerging technologies, as well as the challenges and opportunities associated with these technologies’ integration into PA and health promotion, have been elaborated throughout this Editorial. In this Editorial, the following questions might have been posed: What emerging technologies will we use five to ten years from now to promote more healthful behaviors? Relatedly, what should we be doing currently to prepare for that future? 

Fortunately, new smart mobile devices and other Internet-based emerging technologies make it possible for all parties (e.g., researchers, healthcare providers, technology companies) to work together in promoting PA and health in powerful and innovative ways that were previously unimaginable. Indeed, many of the cutting-edge studies covered in this article have demonstrated the power of smart mobile devices and Internet-based technologies in transforming PA assessment and intervention. Akin to the changing landscape of healthcare over the past decade from reactive to preventive forms of treatment, we can build our own Internet of Health Things with technology and PA to attenuate and prevent chronic diseases. For example, we can connect PA specialists with clients via smart devices, such as health wearable devices, smartphones, and wireless weight scales, among other technologies, of which these clients possess. Specifically, we would have the potential to provide clients with access to their own real-time health data (e.g., weight taken by a wireless scale, blood pressure taken with a wireless blood pressure cuff) via mobile device apps while using a health wearable device to monitor PA. The preceding data could then be uploaded daily to the Internet via a secure server to healthcare providers. On the basis of the PA specialists’ review of a client’s real-time health data, healthcare professionals could then proactively develop well-designed, personalized exercise prescriptions to improve clients’ health and wellness—offering external incentives to clients for staying physically active and adhering to exercise protocols [34].

It should be noted, however, that technological advancement and the prevalence of chronic diseases are currently driving the development of thousands of apps, devices, sensors, wearables, and Internet-based tools. Despite the well-meaning reasons behind organizations and entities’ development of these products, research into the effectiveness of many of these emerging technologies is still in its infancy. For instance, little longitudinal data is available concerning how successful these emerging technologies are at enabling users to lose weight, get fit, or sleep better. Further, how emerging technologies empower clients to change health behaviors (e.g., participate in greater PA) and become healthier remains largely unexplored. Therefore, while pursuing this area of inquiry, it is important for researchers and healthcare professionals to keep in mind that improved technology, data, and connectivity will not promote PA and health per se. Rather, emerging technologies and their applications have the potential to enable users to change behavior and work more effectively when healthcare professionals take care to implement a PA and health intervention using sound theoretical backing and proper intervention fidelity measures. 

Taken together, it is important to note that careful consideration of these challenges and opportunities set the stage for transformative approaches to scientific discovery and effective intervention implementation in the field of PA and health. Indeed, technology is continuously changing our lives, with the use of these technologies in the promotion of PA taking place most frequently over the last decade. As technology has become more advanced, however, these emerging technologies offer numerous exciting opportunities to assess and promote PA from an innovative paradigm when properly implemented to change individuals’ PA behaviors. That is, to effectively assess and promote PA using emerging technology, we need to take into consideration the design, cost, and behavioral theories, as well as build partnerships with the technology industry, communities, interdisciplinary teams, and other relevant public sectors. In this manner, the promise of emerging technology in the facilitation of a more physically active and healthy global population can be more fully realized.

## 8. Directions for Future Research

Given the limitations in the empirical studies with emerging technology, researchers and healthcare professionals have a number of questions to answer prior to successfully and effectively promoting PA and health through advanced technologies. To begin, some recommendations for future exergaming research are as follows: (1) examine the long-term efficacy of exergaming use in non-structured home settings for PA promotion using high-quality randomized controlled trials, and the potential benefits of family/group play and potential barriers in such settings; (2) determine whether individuals with access to exergaming actually replace their screen time with exergaming as opposed to traditional sports or PA; (3) conduct process evaluation for exergaming to ensure the intervention fidelity; (4) investigate exergaming use in early childhood and their subsequent effectiveness; (5) ascertain the effectiveness of using a multi-player mode in comparison to single-player mode; (6) examine the effectiveness of home-based, patient-implemented exergaming rehabilitation as compared with clinic-based rehabilitation research; (7) quantify the role of exergaming in contributing to individual’s daily PA levels; (8) implement serious games or storytelling games that promote PA behaviors; (9) investigate the effects of multiple sports-based exergaming and different exergaming consoles on specific motor skills; and (10) investigate the extent to which exergaming can promote players’ learning and maintenance of new movement and cognitive skills [48,49,50,51].

The fact that online social media-based PA interventions are in their infancy, have small sample sizes, and low study power, make it difficult to discern group differences. Further, some studies lacked objective PA assessments, which limited the validity of the assessed PA outcomes [52]. Therefore, future research should implement true experimental designs that objectively measure PA with larger sample sizes. We should also further examine the long-term health effects of short-term interventions—particularly among older youth populations using mobile health apps with social media features. When it comes to mobile health apps, the validity and reliability of these apps that can track PA and physiological variables must be further evaluated [53].

There are challenges to the implementation of health wearables in real-world settings. First, few works of literature exist confirming health wearables’ efficacy in free-living conditions, and thus, more research is needed to compare the health metric information of commercially-available health wearables to research-grade health wearables [54]. Second, persuading previously sedentary individuals to track their PA is another challenge—particularly as the initial novelty of such technologies wanes. Third, health wearables must be affordable for all so that PA can be tracked by various target populations, with such information subsequently shared with healthcare professionals and used in the development and implementation of the health promotion plan. Hence, promoting PA and reducing sedentary behaviors with affordable wearables among various populations becomes a hot topic in the field. 

In light of the research on virtual reality and PA, no large scale, methodologically-rigorous studies have been completed. Due to its infancy, there is a need for more PA intervention research to prove virtual reality’s efficacy in promoting PA and health among healthy and clinical populations. Therefore, the following research questions might be considered for future studies: (1) how might we convince the clients to understand the potential of virtual reality on health outcomes given the costliness of virtual reality programs? (2) Once implemented, how might virtual reality serve as an effective tool to promote PA and health [55]? And (3) how to sustain virtual reality programs in the long run? In addition, as the technology progresses, augmented reality games, such as Pokémon Go deserve further examination [34]. For example, it is important to examine the efficacy and effectiveness of augmented reality games such as Pokémon Go on individuals’ PA behavior and health outcomes through rigorous experiment design, particularly on youth and young adults. Baranowski [56] has provided a series of questions for future research and directions, such as: “Was playing it with friends (cooperators or competitors) critical to the experience?” and “Were some aspects of the game more played or more effective, or did different aspects (e.g., different characters, different locations) motivate different individuals?” Finally, some GPS/GIS-based games, such as Geocaching, may be effective for changing PA behaviors, and the safety of this game will likely turn off users [57]. The research questions concerning the use of GPS/GIS-based programs for PA and health promotion include but are not limited to: (1) how can GPS/GIS produce reproducible results for PA intensity and type? (2) What can be done if the players lose the GPS signal for prolonged periods of time? and (3) can this new technology seamlessly integrate into one’s daily life [34]?

## 9. Summary

In summary, technology is continuously evolving and constantly changing our lives. On the horizon are novel and exciting cutting-edge technologies that have great potential for PA promotion. Indeed, human beings have applied technology in promoting PA for some time. Yet, in this day and age, emerging technologies and relevant behavioral theories are providing us with needed and exciting opportunities to assess and promote PA on a larger scale—particularly when discussing novel technologies and methodologies such as augmented reality games, crowdsourcing, and online active gaming. Undoubtedly, the technology era appears to be a prime time for researchers and healthcare professionals in PA and health. Offering a logical and clear critique of emerging technologies in PA and health promotion, we sincerely hope that the special issue, along with this editorial, can provide useful suggestions and practical implications for researchers, practitioners, and educators in the fields of public health, kinesiology, PA and health, and healthcare.
